# Skin Microbiota Variation Among Bat Species in China and Their Potential Defense Against Pathogens

**DOI:** 10.3389/fmicb.2022.808788

**Published:** 2022-03-31

**Authors:** Zhongle Li, Aoqiang Li, Wentao Dai, Haixia Leng, Sen Liu, Longru Jin, Keping Sun, Jiang Feng

**Affiliations:** ^1^Jilin Provincial Key Laboratory of Animal Resource Conservation and Utilization, Northeast Normal University, Changchun, China; ^2^College of Animal Science and Technology, Jilin Agricultural University, Changchun, China; ^3^Key Laboratory of Vegetation Ecology, Ministry of Education, Changchun, China; ^4^College of Life Sciences, Henan Normal University, Xinxiang, China

**Keywords:** bat, *Pseudogymnoascus destructans*, pathogen defense, skin microbiota, white-nose syndrome

## Abstract

Host-associated skin bacteria are essential for resisting pathogen infections and maintaining health. However, we have little understanding of how chiropteran skin microbiota are distributed among bat species and their habitats, or of their putative roles in defending against *Pseudogymnoascus destructans* in China. In this study, we characterized the skin microbiomes of four bat species at five localities using 16S rRNA gene amplicon sequencing to understand their skin microbial composition, structure, and putative relationship with disease. The alpha- and beta-diversities of skin microbiota differed significantly among the bat species, and the differences were affected by environmental temperature, sampling sites, and host body condition. The chiropteran skin microbial communities were enriched in bacterial taxa that had low relative abundances in the environment. Most of the potential functions of skin microbiota in bat species were associated with metabolism. Focusing on their functions of defense against pathogens, we found that skin microbiota could metabolize a variety of active substances that could be potentially used to fight *P. destructans*. The skin microbial communities of bats in China are related to the environment and the bat host, and may be involved in the host’s defense against pathogens.

## Introduction

Animal skin is the first line of defense against pathogen invasion and carries diverse symbiotic microbiota that affect host development, reproduction, and health (Braendle et al., 2003; Koropatnick et al., 2004; Jani and Briggs, 2018). Meanwhile, the host, environment, and pathogens also affect the development of host-associated skin microbiomes (Bates et al., 2018; Jiménez et al., 2019). Thus, uncovering patterns in the distribution of skin bacterial communities across host species and their environments could help increase our understanding of how host-associated bacteria are distributed, and their potential roles in defending against pathogens.

The host species is generally a valuable predictor of skin microbiome structure (Jiménez et al., 2019; Kruger, 2020). In 38 mammalian species, host order and species had the most significant influences on skin microbial communities (Ross et al., 2018). For closely related species, such as congeners, the microbial communities also differed, but with relatively smaller variation (Muletz-Wolz et al., 2018; Jiménez et al., 2019). Different sympatric host species exhibited different bacterial community composition, even when they shared the same environment (Kueneman et al., 2014; Muletz-Wolz et al., 2018). These studies suggest that the evolutionary history, as well as the biological and chemical traits of hosts, might be predominant influences on the composition of skin-associated bacteria.

Environment is another important factor influencing skin microbial community structure due to continuous skin contact with the external environment (Loudon et al., 2014; Avena et al., 2016). Massive numbers of bacteria exist in the environments, and they provide a reservoir for host skin microbiota and the possibility of bacterial transmission from the environment to the hosts (Muletz et al., 2012; Ross et al., 2019). Hosts can also select some specific bacteria to colonize their skin (Walke et al., 2014; Rebollar et al., 2016). Additionally, environmental conditions, such as temperature and salinity, might affect the community structure of host-associated microorganisms (Lokmer and Mathias Wegner, 2015; Hughey et al., 2017). Therefore, investigations along environmental gradients could clarify how environmental characteristics affect host-associated microbial communities.

An invading pathogen may damage the host skin microbiome (Stecher et al., 2007; Barman et al., 2008). For instance, the deadly fungus *Batrachochytrium dendrobatidis* disturbed the amphibian skin bacterial community composition and structure in a natural epidemic and in experimental infections (Jani and Briggs, 2014). Previous studies revealed some specific bacterial strains in the skin that inhibited the growth of this pathogen (Woodhams et al., 2015; O’Sullivan et al., 2019). Skin-associated bacteria may produce a variety of active secondary metabolites, including terpenoids, polyketides, alkaloids, and peptides, all of which can protect the host against pathogens (Arasu et al., 2013; Cantrell et al., 2019). However, our understanding of the function of the skin microbial community is still limited, though it is important in host defense against pathogens.

Previous studies have focused on the skin microbiome in animals such as corals, sponges, amphibians, and humans (Rosenberg et al., 2007; Giles et al., 2013; Byrd et al., 2018; Jani and Briggs, 2018). There has been an emphasis on the factors shaping host-associated microbiomes and host defense against pathogens (Krediet et al., 2013; Lemieux-Labonté et al., 2017). However, studies on the skin microbiota of bats are limited but increasing, especially considering the rich diversity and global distribution of chiropterans (Lemieux-Labonté et al., 2016; Winter et al., 2017; Fountain et al., 2019; Grisnik et al., 2020). As we know, over the past decades, a psychrophilic fungus *Pseudogymnoascus destructans* (Gargas et al., 2009) has invaded chiropteran hair follicles, sebaceous glands, and apocrine glands (Meteyer et al., 2009). It causes white-nose syndrome (WNS) and has killed millions of bats across North America (Lorch et al., 2016). Usually, the *P. destructans* load and infection prevalence reach maximum levels during late hibernation (Langwig et al., 2015). It has been proposed that some symbiotic skin bacteria can inhibit the growth of *P. destructans* (Hoyt et al., 2015; Li et al., 2022). Yet, little research on the skin microbiome associated with WNS has been conducted (Avena et al., 2016; Lemieux-Labonté et al., 2017; Ange-Stark et al., 2019; Li et al., 2021; Vanderwolf et al., 2021). In contrast to bats in North America, bats in China have relatively low *P. destructans* loads and infection prevalence, and do not have WNS disease symptoms and mortality (Hoyt et al., 2016a,b). This suggests that bats are resistant to *P. destructans* in China. It is possible that bats have developed intrinsic and adaptive immune responses to *P. destructans* through their long-term coevolution in China. Alternatively, considering that skin microbiota have an important function in defense against pathogens, host-associated bacteria are involved in defending against fungal invasion. One recent study determined the temporal and spatial dynamics of microbe populations and their potential functions on greater horseshoe bats (*Rhinolophus ferrumequinum*) in China (Li et al., 2021). However, the distribution of host-associated bacteria across different host species and how the presence of pathogens influenced it is still not well-understood.

We investigated the skin microbiome of four widely distributed bat species across five localities in China during late hibernation, when bats typically have the greatest fungal load and prevalence (Hoyt et al., 2020). We attempted to clarify variation in bat skin and environmental microbial community composition and structure, and the putative defense mechanism of skin microbiota against pathogens. We had three objectives: (1) elucidate the effects of environment and host on the skin microbiome; (2) clarify geographic variation in the microbiome using *Murina leucogaster* as an example; and (3) predict and compare microbiome putative functions, with an emphasis on those related to pathogen defense.

## Materials and Methods

### Field Sampling

We collected epidermal swabs from four bat species: *Mu. leucogaster* (*n* = 46), *R. ferrumequinum* (*n* = 59), *Myotis petax* (*n* = 17), and *Rhinolophus pusillus* (*n* = 9). We also collected corresponding environment samples (*n* = 35) from five localities: New cave (Jilin Province), Gezi cave (Jilin Province), Di cave (Jilin Province), Temple cave (Liaoning Province), and Water channel (Henan Province). Data was collected at the end of March and the beginning of April in 2018 (Table 1 and Figure 2D). The *R. ferrumequinum* samples used in this research were previously studied by Li et al. (2021). In order to examine the geographic variation in skin microbiomes, we selected *Mu. leucogaster* as an example and collected samples from four sites: Gezi cave, Temple cave, Di cave, and New cave.

**TABLE 1:** Summary of host species, site, and sample sizes information.

Locality (province)	Species	Sampling date	No. of bats sampled	No. of environments sampled	Roosting temperature (mean ± SD)
New cave (Jin Lin)	*Mu. leucogaster*	08/04/2018	13	4	8.65 ± 0.22
New cave (Jin Lin)	*M. petax*	08/04/2018	8	3	9.02 ± 0.78
New cave (Jin Lin)	*R. ferrumequinum*	08/04/2018	20	5	8.64 ± 0.17
Gezi cave (Jin Lin)	*Mu. leucogaster*	09/04/2018	9	3	6.21 ± 0.86
Gezi cave (Jin Lin)	*M. petax*	09/04/2018	9	4	5.29 ± 1.59
Gezi cave (Jin Lin)	*R. ferrumequinum*	09/04/2018	13	5	7.21 ± 0.43
Di cave (Jin Lin)	*Mu. leucogaster*	05/04/2018	11	0	5.8
Temple cave (Liao Ning)	*Mu. leucogaster*	06/04/2018	13	0	5.51 ± 1.61
Temple cave (Liao Ning)	*R. ferrumequinum*	06/04/2018	16	5	7.94 ± 0.43
Water channel (He Nan)	*R. pusillus*	25/03/2018	9	3	10.44 ± 0.51
Water channel (He Nan)	*R. ferrumequinum*	24/03/2018	10	3	8.19 ± 0.42

**FIGURE 1:**
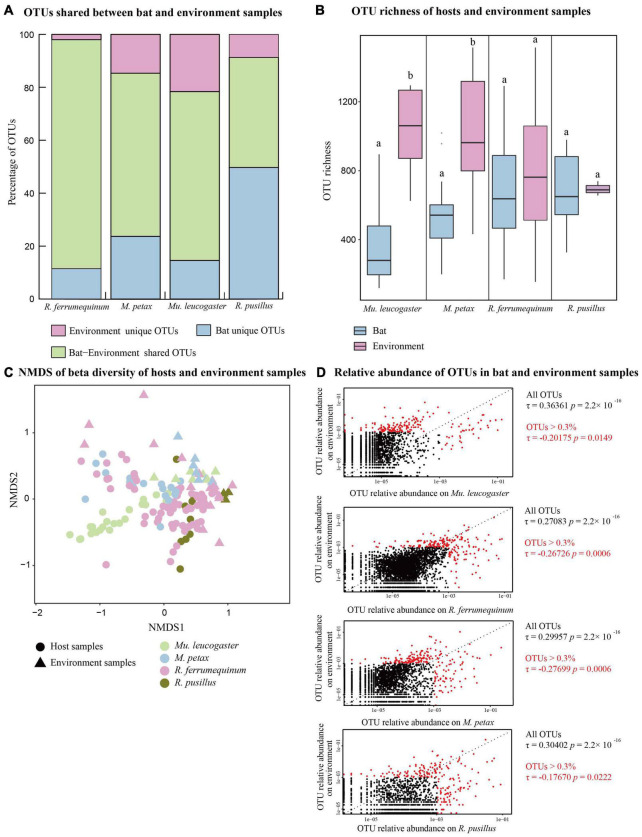
Bat skin and environment bacterial communities of four bat species across five sites. **(A)** Percentage of shared and unique Operational Taxonomic Units (OTUs) of each bat species and environment samples. **(B)** OTU richness of hosts and environment samples. Letters represent significant differences among groups. **(C)** Beta diversities of bat and environment samples. Non-metric multidimensional scaling analysis of Bray Curtis distances. **(D)** Relative abundances of OTUs on each bat species and its corresponding environment samples. Red dots show OTUs with a total relative abundance >0.3%. Kendall’s ranked correlations, *R*_Tau_, and *P*-values are shown to the right of each plot.

**FIGURE 2:**
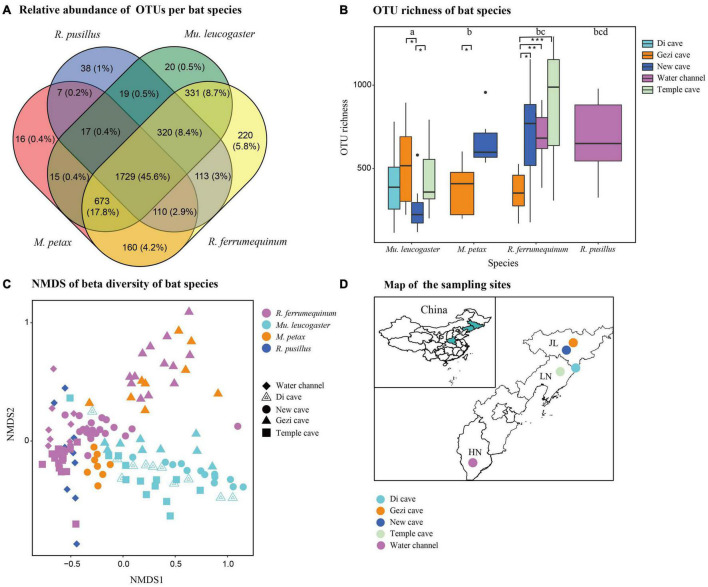
Skin bacterial community structure of four bat species from five sites. **(A)** Relative abundance of shared and unique Operational Taxonomic Units (OTUs) on each bat species. **(B)** OTU richness of four bat species. Letters and * represent significant differences among groups. *P < 0.05, **P < 0.01, ***P < 0.001. **(C)** Beta diversity of four bat species. Non-metric multidimensional scaling analysis of Bray Curtis distances. **(D)** Map of the sampling sites in China.

We dipped sterile polyester swabs in sterile water and wiped five times along the bats’ forearms and muzzles, as previously described (Langwig et al., 2015), to test for *P. destructans*. Bat skin bacterial samples were collected with a similar method, wiping the microbes in the bats’ entire dorsal surface of the wing membrane. Environment samples were also obtained by swabbing the cave walls under the bats (∼5 cm) five times using linear strokes. These samples were stored in 500 μl RNAlater (TIANGEN, Beijing, China) tubes at −20°C within 24 h of sampling until returning to the laboratory, where samples were stored in −80°C refrigerator for DNA extraction. We measured bat roosting temperatures using an infrared thermometer (Fluke, 62 MAX IR, Everett, WA, United States). After swabbing, we measured bat forearm length and weight and calculated the body mass index (BMI = weight/forearm length) and identified the sex and species. Bats were released immediately after sampling. Sampling was performed by the same person to limit individual differences in swabbing techniques. All studies were approved by the Laboratory Animal Welfare and Ethics Committee of Jilin Agricultural University.

### *Pseudogymnoascus destructans* Test

Fungal DNA was extracted from swabs using modified DNeasy Blood and Tissue kits (Qiagen, Hilden, Germany). The modified protocol included the use of lyticase during the lysis step (Hoyt et al., 2016a). Each DNA extraction plate contained seven negative controls (blanks) and one positive control derived from a *P. destructans* ATCC MYA-4855 isolate (Ingala et al., 2017). We used real-time quantitative PCR (qPCR) to examine *P. destructans* following the method of Muller et al. (2013). All samples were run in duplicate. Serial dilutions of the positive control, from 2 ng/μl to 2 fg/μl, were prepared and analyzed with qPCR to calculate fungal loads (*P. destructans* load = 10^[(Ct −22.04942)/−3.34789] (Langwig et al., 2015).

### Bacterial DNA Extraction, Processing, and Sequencing

Genomic DNA was extracted from swabs using the E.Z.N.A™ Mag-Bind Soil DNA Kit (OMEGA Bio-Tek, Norcross, GA, United States) according to manufacturer protocol. PCR amplification was used on the first pair of primers (universal 341F/805R) to target the V3–V4 region of the 16S rDNA and contained 17 bp barcodes (Lu et al., 2015). The PCR reaction system and conditions have been described (Li et al., 2021). The amplification products were purified with the Agencourt AMPure XP beads (Beckman Coulter, Brea, CA, United States). A Qubit 2.0 DNA Assay Kit (Life Technologies, Carlsbad, CA, United States) was used to check the DNA concentration of each sample. PCR amplicons of all samples were sequenced with paired-end 2 × 300 bp on the Illumina MiSeq platform at Sangon Biotech Co., Ltd. (Shanghai, China).

We amplified 9,355,062 raw sequences from the 131 swabs of bat species and 35 corresponding environment samples. The number of reads in the bat samples was 7,311,640 [average 55,814 reads/sample (range of 36,784–89,591)], and the number of reads in the environment samples was 2,043,422 [average 58,383 reads/sample (range of 35,835–91,204)]. We used Cutadpat to remove adapter sequences from the high-throughput sequencing reads (Martin, 2011). Quantitative Insights Into Microbial Ecology (QIIME v 1.9.1) was used to process the forward and reverse reads based on the 10 bp barcode (Caporaso et al., 2010). High-quality reads met the following parameters: they contained no N character sequences, no errors in barcode sequence, and a minimum of five consecutive base pairs (*Q* ≥ 20). After quality processing, a total of 6,705,497 (average 51,187 reads/sample) and 1,940,539 reads (average 55,443 reads/sample) were obtained from bat and environment samples, respectively. Sequences were processed using VSEARCH v. 7.0.1234 (Edgar, 2010) and then clustered at the ≥97% similarity threshold to generate an Operational Taxonomic Unit (OTU) database, with singletons and chimeric sequences excluded during this process. Taxonomy was assigned using the RDP classifier and Greengenes database (DeSantis et al., 2006; Cole et al., 2007). The OTU table was filtered using a minimum cluster size of 0.001% of the total reads to improve accuracy (Bokulich et al., 2013). The OTUs classified as mitochondrial and chloroplastic were removed. Finally, the OTU table was rarefied according to the sample with the lowest number of reads. The final rarefied OTU table, including bat and environment samples, had 3,818 OTUs.

### Data Analysis

We clarified the relationship between host skin microbiota and corresponding environment microbiota using the content of shared and unique OTUs. The alpha diversity (OTU richness and Shannon diversity) of skin microbial diversity of host and environment samples was calculated. *T*-tests and Wilcoxon tests were used to compare alpha diversities between host species and their environment samples. Beta diversity was calculated with Bray Curtis distance matrices and plotted using non-metric multidimensional scaling (NMDS) in *k* = 2 dimensions using the *ordinate()* and *plot_ordination()* functions in the R package *phyloseq* (McMurdie and Holmes, 2013). Permutational multivariate analysis of variance (PERMANOVA) was used to test the significance of beta diversity between bat and environment samples using the *adonis()* function in the *vegan* package (Oksanen et al., 2015). Kendal’s tau ranked correlations of OTU relative abundance were calculated between host species and their environment samples (Fitzpatrick and Allison, 2014). We used all OTUs and OTUs with a relative abundance greater than 0.3% to calculate the correlations of host and environment samples.

We determined whether the skin microbiota differed among the individual host species from multiple sites. We used a Venn diagram to represent the relative abundance of shared and unique OTUs among the host species. For alpha diversity, OTU richness and Shannon diversity were calculated, and Kruskal-Wallis tests were used to determine differences across host species. We used generalized linear models (GLM) with a quasi-Poisson distribution to examine variation depending on factors such as sites, BMI, temperature, sex, *P. destructans* infection status, and *P. destructans* load in OTU richness and Shannon diversity. The calculation and visualization of beta diversity among host species and sites was as described above. To assess the influence of each explanatory variable (BMI, temperature, sex, *P. destructans* infection status and *P. destructans* load) on host species microbiota composition, we used partial distance-based redundancy analysis (db-RDA) while controlling for the host and site variables. Adjusted R-squared (*R*^2^) values were calculated to clarify the explanatory variables (Starcher, 1942). Significance of partial db-RDA was tested via 9,999 permutations with the *anova.cca()* function of the *vegan* package. The unweighted pair group method with arithmetic mean (UPGMA) was used on Bray Curtis distances of mean >0.1% for OTU relative abundances at the genus level to determine clustering patterns across host species. We performed generalized linear mixed models (GLMMs) to assess OTUs that were differentially abundant between species in the glmmTMB package using a beta distribution and a logit link (Brooks et al., 2017). In this model, the OTU and species were set as fixed effects, and site was included as a random effect.

Functional prediction was conducted using Phylogenetic Investigation of Communities by Reconstruction of Unobserved States2 (PICRUSt2; Douglas et al., 2020) from the 16S rRNA gene sequences (Langille et al., 2013; Beaumont et al., 2021). The predicted functional gene abundance was based on the OTU table. The obtained prediction results can be used to classify gene families by KEGG Orthology (Wei et al., 2020). The linear discriminant analysis (LDA) size effect (LEfSe) was carried out to determine the significant differences in KEGG pathways across bat species (Segata et al., 2011), with an LDA score >2.0 considered as a cut-off (Clemente et al., 2015). We explored gene relative abundance differences among species from two functional classes associated with bacterial defense mechanisms: metabolism of terpenoids and polyketides (MTP) and biosynthesis of secondary metabolites (BSM) (Rebollar et al., 2018). Kruskal-Wallis tests and ANOVA were used to analyze the significant differences of gene relative abundance among the bat species.

To evaluate the geographic variation in bacterial community structure in *Mu. leucogaster*, we compared alpha- (OTU richness and Shannon diversity) and beta-diversities across four sites. We used indicator Value tests (*IndVal*) (Dufrêne and Legendre, 1997) to identify the most representative OTUs at genus levels with a relative abundance >1% on *Mu. leucogaster* across sites. The *IndVal* values were computed in the package *indicspecies* accessed by the *multipatt()* function (De Cáceres and Legendre, 2009), and significance was assessed with 9,999 permutations. OTUs with *IndVal* values ≥0.4 were considered to be indicators (Lemieux-Labonté et al., 2017). Kendal’s tau ranked correlations of OTU relative abundance >0.1% were calculated on *Mu. leucogaster* across sites. We used LEfSe analysis to evaluate the significant differences in KEGG pathways on *Mu. leucogaster* across sites. Kruskal-Wallis tests and ANOVA were used to detect gene relative abundances of BSM and MTP in *Mu. leucogaster*. All statistical analyses were performed in R version 4.0.5 (R Core Team, 2019).

## Results

### *Pseudogymnoascus destructans* Infection

The fungal loads were significantly different among the four bat species (Kruskal-Wallis test, Chi-squared = 30.245, *P* < 0.001). *Myotis petax* had the highest *P. destructans* infection intensity (average log-transformed *P. destructans* loads: −2.62), followed by *R. ferrumequinum* (−4.20), *Mu. leucogaster* (−4.24), and *R. pusillus* (−6.11) (Supplementary Figure 1A). The highest *P. destructans* prevalence was on *M. petax* (Supplementary Figure 1B). Among different localities for *Mu. leucogaster*, *P. destructans* loads were the lowest in Temple cave (−5.28) (ANOVA, *F* = 12.6, *P* < 0.001), and the highest *P. destructans* prevalence was in Gezi cave (−3.69) (Supplementary Figure 1B).

### Microbial Communities Between Host and Environment

The majority of the OTUs on bat skin were also present in the environment, with the exception of *R. pusillus* (Figure 1A). The OTU richness was significantly lower than the environment samples in *M. petax* and *Mu. leucogaster* (*M. petax*: *t* = −2.99, *P* = 0.018; *Mu. leucogaster*: *w* = 5, *P* = 0.003), but similar in *R. ferrumequinum* (*t* = −0.84, *P* = 0.406) and *R. pusillus* (*t* = −0.14, *P* = 0.887) (Figure 1B). The Shannon diversity was also significantly lower than the environment samples in *M. petax* and *Mu. leucogaster* (*M. petax*: *t* = −3.11, *P* = 0.014; *Mu. leucogaster*: *w* = 0, *P* < 0.001), but similar in *R. ferrumequinum* (*w* = 432, *P* = 0.236) and *R. pusillus* (*w* = 22, *P* = 0.145) (Supplementary Figure 2A). All hosts combined, or each host separately, had significantly different microbial structures in comparison with corresponding environment samples (all hosts: NMDS with stress = 0.18; PERMANOVA, Pseudo-*F*_1,141_ = 13.19, *P* = 0.001, *R*^2^ = 0.173; *R. ferrumequinum*: PERMANOVA, Pseudo-*F*_1,76_ = 7.07, *P* = 0.001, *R*^2^ = 0.086; *M. petax*: PERMANOVA, Pseudo-*F*_1,23_ = 2.33, *P* = 0.007, *R*^2^ = 0.096; *Mu. leucogaster*: PERMANOVA, Pseudo-*F*_1,28_ = 11.11, *P* = 0.001, *R*^2^ = 0.292; *R. pusillus*: PERMANOVA, Pseudo-*F*_1,11_ = 3.36, *P* = 0.008, *R*^2^ = 0.251, Figure 1C).

Each bat species was positively correlated with the relative abundances of OTUs in its corresponding environment samples (Figure 1D), with *Mu. leucogaster* having the highest correlation (*Mu. leucogaster*: *R*_τ_ = 0.364, *P* < 0.001; *R. ferrumequinum*: *R*_τ_ = 0.271, *P* < 0.001; *M. petax*: *R*_τ_ = 0.300, *P* < 0.001; *R. pusillus*: *R*_τ_ = 0.304, *P* < 0.001). When we calculated ranked correlations with the most relatively abundant OTUs (OTUs > 0.3%), all of the correlations between bat and environment samples became significantly negative (Figure 1D; *Mu. leucogaster*: *R*_τ_ = −0.202, *P* = 0.014; *R. ferrumequinum*: *R*_τ_ = −0.267, *P* = 0.001; *M. petax*: *R*_τ_ = −0.277, *P* = 0.001; *R. pusillus*: *R*_τ_ = −0.177, *P* = 0.022) (Figure 1D).

### Structure and Function of Skin Bacterial Communities Across Hosts

Host species shared the majority of the relative abundances of OTUs on skin bacterial communities in all four species (45.6%). Few OTUs unique to individual host species were found (ranging from 0.4% for *M. petax* to 5.8% for *R. ferrumequinum*) (Figure 2A). OTU richness was significantly different from host species at all sites (Kruskal-Wallis: Chi-squared = 29.54, *P* < 0.01; Figure 2B). In the GLM analysis, we found that sites (Chi-squared = 17.52, *P* = 0.001) and BMI (Chi-squared = 5.59, *P* = 0.018) were the significant factors associated with changes in OTU richness. Temperature, sex, *P. destructans* infection status, and fungal load did not significantly affect alpha diversity measures (all *P* > 0.05). Shannon diversity was also significantly different from host species across all sites (Kruskal-Wallis: Chi-squared = 30.14, *P* < 0.01; Supplementary Figure 2B). In the GLM analysis, we found that sites (Chi-squared = 22.6, *P* = 0.001) and BMI (Chi-squared = 5.37, *P* = 0.02) were the significant factors associated with changes in Shannon diversity.

Beta diversity analysis indicated that skin bacterial communities significantly differed among the four bat species (NMDS with stress = 0.17; Bray-Curtis, PERMANOVA, Pseudo-*F*_3,130_ = 3.41, *P* = 0.001, *R*^2^ = 0.074; Figure 2C). Site was also a significant predictor of skin bacterial communities on bat species (Bray-Curtis, PERMANOVA, Pseudo-*F*_3,130_ = 4.11, *P* = 0.001, *R*^2^ = 0.115). The partial db-RDA analysis showed that bat roosting temperature was significantly correlated with microbial composition changes (Bray-Curtis, PERMANOVA, Pseudo-*F*_3,62_ = 2.41, *P* = 0.002, *R*^2^ = 0.011) when we controlled for the host species and sites. Likewise, *P. destructans* infection status, fungal load, BMI, and sex did not significantly affect the beta diversity measures (all *P* > 0.05). All 21 OTUs were differentially abundant among the bat species (GLMMs, *P* < 0.05, Table 2). *M. petax* had communities dominated by the genera *Citrobacter* and families Micrococcaceae and Pasteurellaceae. *R. ferrumequinum* was dominated by OTUs from the genera *Brackiella*, *Corynebacterium*, and *Staphylococcus.* However, *R. pusillus* had relatively fewer dominant OTUs from the genera *Mycobacterium* and *Kaistobacter*. *Mu. leucogaster* had a rich complement of dominant OTUs, such as *Pseudomonas*, *Sphingobacterium*, and *Flavobacterium* (Table 2).

**TABLE 2:** Heatmap of the average relative abundance (>1%) of bacterial Operational Taxonomic Units (OTUs) that were differentially abundant among bat species sampled from different localities.

OTU ID	Taxa	MULE New cave	MULE Di cave	MULE Temple cave	MULE Gezi cave	RHPU Water channel	RHFE Gezi cave	RHFE New cave	RHFE Water channel	RHFE Temple cave	MYPE Gezi cave	MYPE New cave
Otu2	f__Micrococcaceae	0.016	0.139	0.035	0.051	0.004	0.007	0.011	0.018	0.005	0.239	0.241
Otu35	*Citrobacter*	0.000	0.001	0.002	0.001	0.002	0.002	0.007	0.001	0.001	0.002	0.058
Otu89	f__Pasteurellaceae	0.000	0.000	0.000	0.000	0.000	0.000	0.000	0.000	0.000	0.014	0.005
Otu10	*Brackiella*	0.029	0.039	0.032	0.020	0.014	0.080	0.050	0.046	0.029	0.004	0.002
Otu5	*Corynebacterium*	0.000	0.000	0.000	0.000	0.000	0.016	0.155	0.020	0.003	0.000	0.000
Otu12	*Staphylococcus*	0.001	0.000	0.006	0.001	0.002	0.096	0.025	0.009	0.006	0.002	0.001
Otu251	*Mycobacterium*	0.000	0.000	0.000	0.000	0.019	0.000	0.000	0.000	0.000	0.000	0.000
Otu73	*Kaistobacter*	0.000	0.000	0.001	0.001	0.012	0.000	0.002	0.006	0.009	0.001	0.004
Otu4	*Pseudomonas*	0.077	0.092	0.094	0.238	0.004	0.104	0.059	0.107	0.027	0.112	0.097
Otu1	*Pseudomonas*	0.068	0.088	0.150	0.123	0.003	0.118	0.119	0.036	0.018	0.073	0.006
Otu7	*Sphingobacterium*	0.101	0.079	0.048	0.063	0.000	0.019	0.008	0.001	0.001	0.022	0.025
Otu13	f__Brucellaceae	0.069	0.093	0.018	0.013	0.002	0.002	0.002	0.000	0.000	0.003	0.006
Otu3	*Flavobacterium*	0.080	0.073	0.056	0.020	0.004	0.002	0.003	0.001	0.001	0.002	0.044
Otu20	*Myroides*	0.030	0.011	0.042	0.067	0.000	0.025	0.011	0.000	0.010	0.017	0.002
Otu11	f__Intrasporangiaceae	0.059	0.054	0.014	0.059	0.001	0.003	0.004	0.001	0.002	0.006	0.008
Otu15	*Arthrobacter*	0.016	0.052	0.034	0.046	0.002	0.009	0.014	0.011	0.013	0.028	0.019
Otu19	*Sphingobacterium*	0.035	0.000	0.051	0.001	0.000	0.000	0.000	0.000	0.000	0.000	0.001
Otu22	*Flavobacterium*	0.004	0.021	0.040	0.019	0.001	0.008	0.002	0.003	0.007	0.030	0.003
Otu36	*Salinibacterium*	0.017	0.012	0.005	0.030	0.001	0.001	0.005	0.000	0.001	0.004	0.003
Otu23	*Gluconacetobacter*	0.018	0.021	0.018	0.006	0.001	0.001	0.009	0.002	0.003	0.002	0.022
Otu28	*Brevibacterium*	0.022	0.001	0.016	0.002	0.000	0.001	0.002	0.000	0.001	0.000	0.001

*All 21 OTUs were differentially abundant from four bat species in different sites (GLMMs < 0.05). The colors represent the degree of abundance, with warm colors indicating higher abundances. MULE, Mu. leucogaster; RHPU, R. pusillus; RHFE, R. ferrumequinum; MYPE, M. petax.*

**FIGURE 3:**
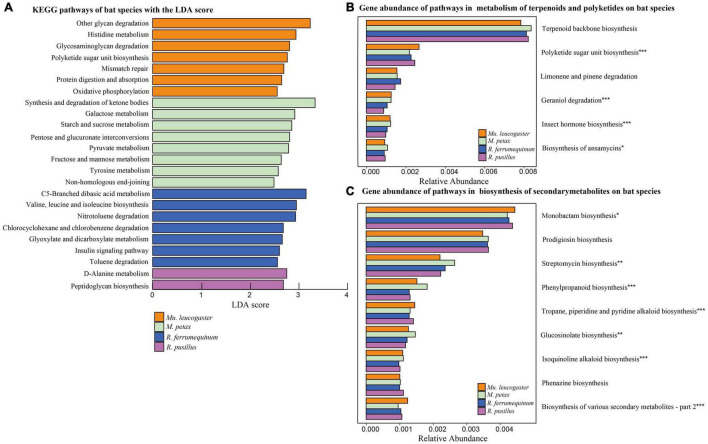
Functional gene predictions. **(A)** KEGG pathways of bat species based on LEfSe analysis. KEGG pathways with the highest linear discriminant analysis (LDA) scores. Gene abundance of pathways within the metabolism of terpenoids and polyketides **(B)** and biosynthesis of secondary metabolites **(C)** on bat species. The gene relative abundances of pathways >0.1%. Asterisks indicate significant difference among groups. *P < 0.05, **P < 0.01, ***P < 0.001.

We detected significant differences (LEfSe analysis: LDA score > 2, *P* < 0.05) in gene relative abundances from KEGG Orthology groups (KOs) on chiropteran skin microbiomes using PICRUSt2 (Figure 3A). Most of the KOs were related to metabolism, followed by genetic information processing, organic systems, and environmental information processing (Supplementary Table 1). For two bacterial defense mechanisms, metabolism of terpenoids and polyketides (MTP) and biosynthesis of secondary metabolites (BSM), 19 out of 21 pathways and 18 out of the 30 pathways described in KEGG were present in bat skin microbiomes, respectively. For those pathways with gene relative abundances >0.1%, different bat species enriched similar pathways but most of them had significant variation across hosts (Figures 3B,C). We analyzed phenazine biosynthesis because of the presence of anti-*P. destructans* active substances in this pathway (Li et al., 2022); however, no significant variation in gene abundance was found among the bat species (Supplementary Table 2).

### Skin Bacterial Community and Function Differences of *Murina leucogaster*

**FIGURE 4:**
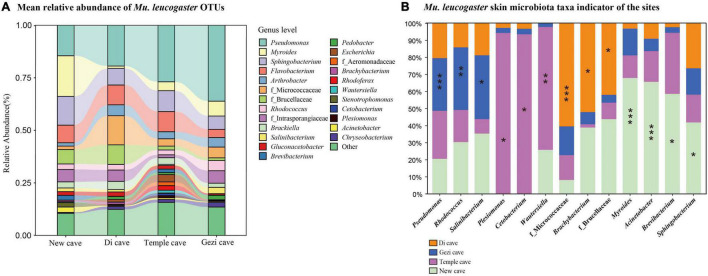
Skin bacterial community composition of *Mu. leucogaster*. **(A)** Alluvial diagram of mean relative abundances of bacterial taxa (genus level) of *Mu. leucogaster* across sites. The relative abundances of bacterial genera > 1%. **(B)** *Mu. leucogaster* skin microbiota taxa indicator of the sites. Significant indicators were found among the 13 most abundant taxa representing more than 1% of total abundance with *IndVal* analysis. Stars indicate significant representative taxa. *P < 0.05, **P < 0.01, ***P < 0.001, *IndVal* ≥ 0.4.

Operational Taxonomic Unit richness of *Mu. leucogaster* was significantly different across sites (Kruskal-Wallis: Chi-squared = 8.17, *P* = 0.01; Figure 2B), while no single factor (temperature, BMI, sex, *P. destructans* infection status or load) was found to significantly affect it (GLM: *P* > 0.05). Shannon diversity of *Mu. leucogaster* was not significantly different across sites (Kruskal-Wallis: Chi-squared = 1.04, *P* = 0.79; Supplementary Figure 2B). Beta diversity of *Mu. leucogaster* skin communities were also consistently different across sites (NMDS with stress = 0.15; Bray-Curtis, PERMANOVA, Pseudo-*F*_3,45_ = 5.50; *P* = 0.001, *R*^2^ = 0.282; Figure 2C), but no factor significantly influenced the bacterial community structure (partial db-RDA analysis, all *P* > 0.05). The mean relative abundance of bacterial taxa on *Mu. leucogaster* differed across sites (Figure 4A). *Pseudomonas* was the dominant genus in Gezi cave (36.1%), Temple cave (26.9%), and Di cave (19.4%). However, the dominant genus in New cave was *Myroides* (19.3%). Multiple indicator taxa were detected at different locations, such as *Salinibacterium*, *Rhodococcus*, and *Pseudomonas* at Gezi cave (Figure 4B). The *P. destructans* loads and relative abundance (>0.1%) of many genera on *Mu. leucogaster* across sites showed a negative correlation, but the results were not statistically significant (data not shown).

LEfSe analysis revealed significant differences in the gene relative abundances from KOs across sites in *Mu. leucogaster* (LDA score > 2, *P* < 0.05), and most KOs were associated with metabolism (Supplementary Figure 3A). In MTP and BSM, except for isoquinoline alkaloid biosynthesis, the gene abundances (>0.1%) of all other pathways were significantly different on *Mu. leucogaster* (Supplementary Figure 4A).

## Discussion

In this study, *P. destructans* loads differed among bat species, and *M. petax* had the highest fungal loads. Typically, *Myotis* were the most commonly infected species with *P.*

*destructans* in Europe (Puechmaille et al., 2011) and North America (Turner et al., 2011). *Myotis petax* typically roosted in cave entrances with their bodies in close contact with the cave wall, similar to the roosting style of *M. lucifugus*, increasing the chance of *P. destructans* infection (Turner et al., 2011). The high fungal loads of *M. petax* were also possibly associated with the immune capacity of the bats. Furthermore, significant differences in fungal loads were detected in *Mu. leucogaster* across different sites, presumably due to the different caves, providing different microclimate for *P. destructans* survival.

The environment affects the community structure and composition of skin microbiota (Manus et al., 2017; Muletz-Wolz et al., 2018). We found that the skin bacterial communities of bat species differed from the corresponding environments in alpha- and beta-diversities (Figures 1B,C), even if they shared the majority of OTUs (Figure 1A). These results are consistent with other studies on bats (Avena et al., 2016; Grisnik et al., 2020), indicating that different bat species share the most bacterial taxa with their corresponding environmental samples at the class level (Avena et al., 2016). In particular, the high abundance of OTUs in bat skin had a negative correlation with corresponding environmental OTUs (Figure 1D), which was also found in Panamanian frogs and *R. ferrumuequinum* (Rebollar et al., 2016; Li et al., 2021). This result suggested that the presence of unique ecological niche-bacterial taxa in skin favored specific growth. However, the cave wall under the bat could not fully reflect the real environmental conditions, so additional environmental samples such as soil and rock should be considered in future studies. In fact, the skin microbial assemblages do not fully reflect the microbiota of the environment, which can be significantly influenced by multiple factors (Ross et al., 2019). For instance, the host may change its skin microbiota for defense against pathogen invasion, even though it is constantly exposed to the external environment (Rosenthal et al., 2011).

Host species is also one of the important factors shaping the skin microbiome (Muletz-Wolz et al., 2018). In this study, skin microbiota varied across bat species, but the different bat species shared the majority of OTUs. This suggested a similar classification of their skin microbiota, which was consistent with results on neotropical bats (Lemieux-Labonté et al., 2016), but the community diversity was different among the bat species (Avena et al., 2016; Lemieux-Labonté et al., 2016). Significant microbial differences in alpha diversity (Figure 2B and Supplementary Figure 2B) and community structure (Figure 2C) were found among the bat species, driven by BMI and temperature. Host body condition makes a significant contribution to skin alpha-diversity in *Eleutherodactylus coqui* (Longo and Zamudio, 2017), and the relative abundance of *Corynebacterium* in human skin microbiota is significantly correlated with BMI (Brandwein et al., 2019). Temperature may indirectly affect microbial community structure by causing changes in host physiology (such as immune function) (Hooper et al., 2012). For humpback whales, variations in the average relative abundance of four core genera of skin microbiome were closely associated with decreasing water temperature (Bierlich et al., 2018). Meanwhile, microorganisms also need suitable temperatures to grow and reproduce. Therefore, temperature becomes a filter of the environment, allowing bacteria with specific characteristics to grow within a certain temperature range, while also potentially increasing the growth of pathogens (Langwig et al., 2015, 2016).

In addition to the variation in skin microbial communities across species, *Mu. leucogaster* had significant geographic differences in OTU richness and community structure (Figures 2B,C). However, the alpha- and beta-diversities were not affected by BMI, temperature, sex, *P. destructans* infection status, and fungal load, implying that sampling position had an important influence on the skin microbiome (Avena et al., 2016). Those differences could be associated with the geomorphology of the caves, such as different space, population size, humidity and temperature. In studies on *M. lucifugus*, local environment was an important factor explaining the pattern of skin microbial communities (Lemieux-Labonté et al., 2017). During hibernation, bats roost on the cave walls for long periods of time, which provides a change for host skin to exchange bacteria with the environment and may select for bacteria with low abundance in the environment (Li et al., 2021). Host genetic factors and other host-intrinsic characteristics also could directly influence the composition of human skin microbiota (Si et al., 2015). Thus, it is also possible that genetic differentiation among bat populations may explain differences in bacterial communities. Further calculation of genetic diversity is needed to elucidate the specific differences.

To better assess bat skin microbiota-pathogen interactions, we analyzed putative functions of the chiropteran microbiome. Many genes were related to metabolic pathways in chiropteran skin microbiota (Figure 3A). Microbial metabolism is a fundamental process of life activity. The metabolism of amino acids and carbohydrates plays an important role in the maintenance of physiological homeostasis and disease defense in hosts (Fuhrer et al., 2010; Thomas et al., 2012). We focused on analyses of the metabolic pathways associated with defense mechanisms, including MTP and BSM (Figures 3B,C). Similar functional classifications of the skin microbiota were found across bat species and sites, suggesting the existence of consistent defense mechanisms.

The MTP class includes degradation pathways such as limonene, pinene, geraniol, and citronellol. *Pseudomonas* and *Rhodococcus* strains have become model organisms for these pathways (Marmulla and Harder, 2014). These two genera inhibit the growth of several conservation relevant fungal diseases, including snake fungal disease, chytridiomycosis, and WNS (Hoyt et al., 2015; Woodhams et al., 2015; Cornelison et al., 2016; Lemieux-Labonté et al., 2017). After *Eptesicus fuscus* is inoculated with *P. destructans*, *Pseudomonas*, and *Rhodococcus* are still the dominant genera in the skin microbiota (Lemieux-Labonté et al., 2020). In this study, *Pseudomonas* had a high abundance in all species except *R. pusillus* (Table 2). Similarly, the skin microbiota of *Mu. leucogaster* in Gezi cave were enriched with a large number of *Pseudomonas*, *Rhodococcus*, and *Salinibacterium* as indicator taxa (Figures 4A,B). The *P. destructans* load and prevalence of *Mu. leucogaster* in Gezi cave were the highest (Supplementary Figures 1A,B), and the OTU richness was also the highest, suggesting the production of more antifungal metabolites (degradation pathways) or more effective competition with the pathogen for resources (Longo et al., 2015). The *Pseudomonas* isolated from *R. ferrumequinum* and *M. petax* in Gezi cave *in vitro* efficiently inhibited the growth of *P. destructans* (Li et al., 2022). The BSM class pathways may be involved in bacterial competition within the community and in protecting the host against pathogens (Rebollar et al., 2016). The highest gene relative abundance of pathways in BSM, across bat species and sites, was involved in monobactam biosynthesis, followed by prodigiosin biosynthesis (Figure 3C and Supplementary Figure 4A). Many novel monobactam derivatives have been evaluated for their antibacterial activities against Gram-negative pathogenic strains, including Aztreonam and BAL30072 compounds (Fu et al., 2016). *Serratia* produces prodigiosin that significantly inhibits the growth of *B. dendrobatidis* and *B. salamandrivorans* (Woodhams et al., 2018). The phenazine-1-carboxylic acid produced by *P. yamanorum* GZD14026 isolated from *M. petax* in Gezi cave effectively inhibited *P. destructans* (Li et al., 2022). These metabolic pathways could be involved in protecting the host against *P. destructans*, leading to asymptomatic bats with a low infection burden.

Our study results demonstrated strong variation of skin bacterial community structure and composition across bat species and sampling sites. The BMI of bats and sampling sites could drive variation in microbial diversity, while environmental temperature and location significantly influenced community structure. However, host skin microbial communities significantly differed from those of the surrounding environments. Among *Mu. leucogaster* populations, strong geographic variation in community structure was found. The putative functions of the bat skin microbiome suggested that secondary metabolite pathways—the MTP and BSM classes—provide potential defense against pathogens and could play a role in protecting bats from the invasion of *P. destructans*. However, the functions of chiropteran skin symbiotic bacteria need further study to fully understand the interactions between microbes, hosts, and pathogens.

## Data Availability Statement

The datasets presented in this study can be found in online repositories. The names of the repository/repositories and accession number(s) can be found in the article/Supplementary Material.

## Ethics Statement

The animal study was reviewed and approved by the Laboratory Animal Welfare and Ethics Committee of Jilin Agricultural University.

## Author Contributions

ZL performed data analysis and drafted the manuscript. ZL, AL, WD, HL, SL, and LJ collected the samples. KS and JF developed the study concept, design, and supervised the project. AL and KS reviewed and revised the manuscript. All authors contributed to the article and approved the submitted version.

## Conflict of Interest

The authors declare that the research was conducted in the absence of any commercial or financial relationships that could be construed as a potential conflict of interest.

## Publisher’s Note

All claims expressed in this article are solely those of the authors and do not necessarily represent those of their affiliated organizations, or those of the publisher, the editors and the reviewers. Any product that may be evaluated in this article, or claim that may be made by its manufacturer, is not guaranteed or endorsed by the publisher.
