# Supplementary material for: Skin Microbiota Variation Among Bat Species in China and Their Potential Defense Against Pathogens
Source: Front Microbiol. 2022 Mar 31;13:808788. doi: 10.3389/fmicb.2022.808788 (PMC9009094; doi:10.3389/fmicb.2022.808788)

**Skin microbiota variation among bat species in China and their potential defense against pathogens**

Zhongle Li^1,2^, Aoqiang Li^1,3^, Wentao Dai^1^, Haixia Leng^1^, Sen Liu^4^, Longru Jin^1^, Keping Sun^1,3,*^, and Jiang Feng^1,2,*^

^1^ Jilin Provincial Key Laboratory of Animal Resource Conservation and Utilization, Northeast Normal University, 2555 Jingyue Street, Changchun, 130117, China

^2^ College of Animal Science and Technology, Jilin Agricultural University, Changchun, 130018, China

^3^ Key Laboratory of Vegetation Ecology, Ministry of Education, Changchun, 130024, China

^4^ College of Life Sciences, Henan Normal University, Xinxiang, 453007, China

* Correspondence to: sunkp129@nenu.edu.cn and fengj@nenu.edu.cn

**Supplementary material**

**Table S1.** KEGG pathways of bat species from LEfSe analysis that had LDA scores > 2.0.

| **Hierarchy level 3** | **Hierarchy level 2** | **Hierarchy level 1** | **Bat species** | **LDA score** | **P value** |
| --- | --- | --- | --- | --- | --- |
| Other glycan degradation | Glycan biosynthesis and metabolism | Metabolism | *Mu. leucogaster* | 3.245 | 5.86E-14 |
| Histidine metabolism | Amino acid metabolism | Metabolism | *Mu. leucogaster* | 2.952 | 7.22E-11 |
| Glycosaminoglycan degradation | Glycan biosynthesis and metabolism | Metabolism | *Mu. leucogaster* | 2.820 | 7.21E-12 |
| Polyketide sugar unit biosynthesis | Metabolism of terpenoids and polyketides | Metabolism | *Mu. leucogaster* | 2.773 | 1.11E-05 |
| Mismatch repair | Replication and repair | Genetic Information Processing | *Mu. leucogaster* | 2.703 | 0.008 |
| Protein digestion and absorption | Digestive system | Organismal Systems | *Mu. leucogaster* | 2.657 | 2.24E-12 |
| Oxidative phosphorylation | Energy metabolism | Metabolism | *Mu. leucogaster* | 2.566 | 1.76E-05 |
| Synthesis and degradation of ketone bodies | Lipid metabolism | Metabolism | *M. petax* | 3.344 | 1.62E-04 |
| Galactose metabolism | Carbohydrate metabolism | Metabolism | *M. petax* | 2.927 | 9.96E-06 |
| Starch and sucrose metabolism | Carbohydrate metabolism | Metabolism | *M. petax* | 2.865 | 2.04E-05 |
| Pentose and glucuronate interconversions | Carbohydrate metabolism | Metabolism | *M. petax* | 2.821 | 5.89E-07 |
| Pyruvate metabolism | Carbohydrate metabolism | Metabolism | *M. petax* | 2.798 | 1.64E-05 |
| Fructose and mannose metabolism | Carbohydrate metabolism | Metabolism | *M. petax* | 2.648 | 5.34E-04 |
| Tyrosine metabolism | Amino acid metabolism | Metabolism | *M. petax* | 2.586 | 1.59E-04 |
| Non-homologous end-joining | Replication and repair | Genetic Information Processing | *M. petax* | 2.500 | 6.77E-05 |
| Ascorbate and aldarate metabolism | Carbohydrate metabolism | Metabolism | *M. petax* | 2.455 | 2.41E-04 |
| Tropane, piperidine and pyridine alkaloid biosynthesis | Biosynthesis of other secondary metabolites | Metabolism | *M. petax* | 2.423 | 1.53E-05 |
| C5-Branched dibasic acid metabolism | Carbohydrate metabolism | Metabolism | *R. pusillus* | 3.160 | 1.56E-09 |
| Valine, leucine and isoleucine biosynthesis | Amino acid metabolism | Metabolism | *R. pusillus* | 2.960 | 6.59E-05 |
| Nitrotoluene degradation | Xenobiotics biodegradation and metabolism | Metabolism | *R. pusillus* | 2.944 | 3.02E-10 |
| Chlorocyclohexane and chlorobenzene degradation | Xenobiotics biodegradation and metabolism | Metabolism | *R. pusillus* | 2.691 | 5.54E-05 |
| Glyoxylate and dicarboxylate metabolism | Carbohydrate metabolism | Metabolism | *R. pusillus* | 2.670 | 0.014 |
| Insulin signaling pathway | Endocrine system | Organismal Systems | *R. pusillus* | 2.612 | 3.86E-09 |
| Toluene degradation | Xenobiotics biodegradation and metabolism | Metabolism | *R. pusillus* | 2.570 | 1.22E-06 |
| ECM-receptor interaction | Signaling molecules and interaction | Environmental Information Processing | *R. pusillus* | 2.397 | 7.30E-08 |
| Retinol metabolism | Metabolism of cofactors and vitamins | Metabolism | *R. pusillus* | 2.349 | 6.23E-05 |
| D-Alanine metabolism | Metabolism of other amino acids | Metabolism | *R. ferrumequinum* | 2.762 | 5.72E-05 |
| Peptidoglycan biosynthesis | Glycan biosynthesis and metabolism | Metabolism | *R. ferrumequinum* | 2.693 | 7.63E-05 |

**Table S2.** Genes involved in the production of anti-*P. destructans* metabolites from bat skin microbiota. BSM, Biosynthesis of secondary metabolites; PCA, Phenazine-1-carboxylic acid.

| KEGG class | KEGG pathway | KO | Gene description | Anti-*P. destructans* metabolite |
| --- | --- | --- | --- | --- |
| BSM | Phenazine biosynthesis | K06998 | phzF; trans-2,3-dihydro-3-hydroxyanthranilate isomerase | PCA |
| BSM | Phenazine biosynthesis | K13063 | phzE; 2-amino-4-deoxychorismate synthase | PCA |

**Figure S1** *P. destructans* infection profiles of bat species across sites. (a) Boxplot of

*P. destructans* infection intensity of bat species across sites (*P* < 0.001). Letters and *

represent significant differences among groups. **P* < 0.05, ***P* < 0.01, ****P* < 0.001. (b) Bar chart of *P. destructans* infection prevalence of bat species across sites.

**
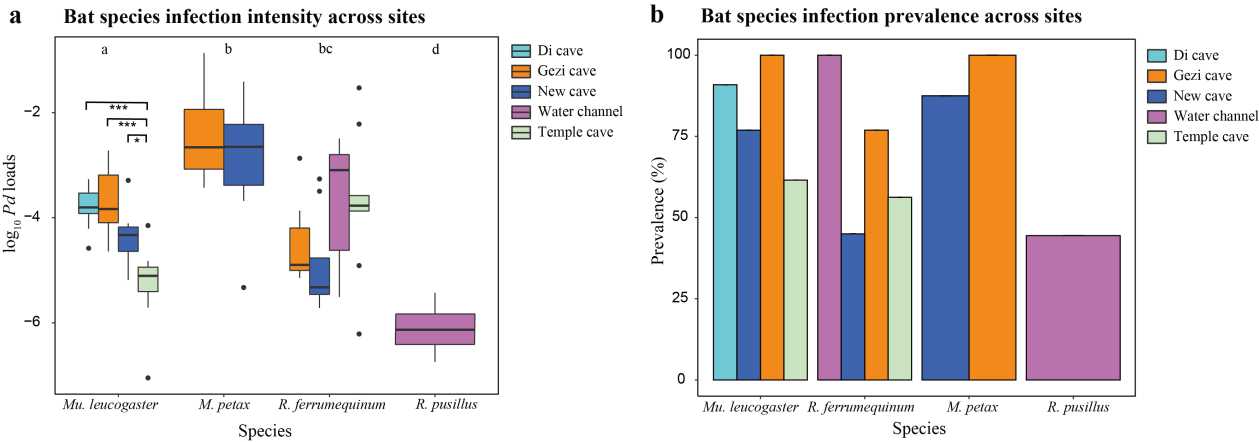
**

**Figure S2** Alpha diversity (Shannon) of bat species and environment samples. (a) Shannon diversity of each host and its corresponding environment. Letters


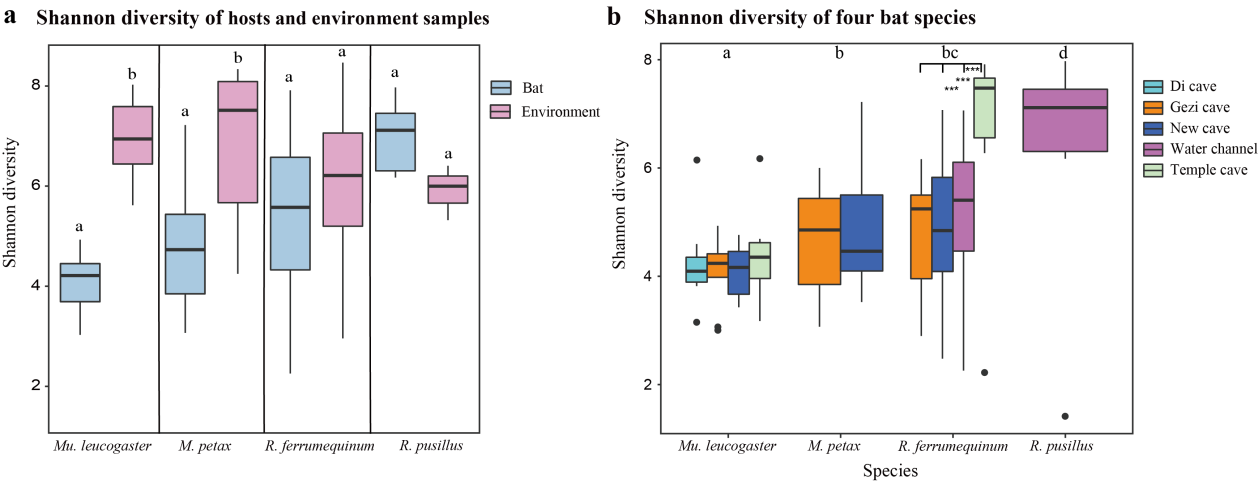
represent significant differences among groups. (b) Shannon diversity of four bat species. Letters and * represent significant differences among groups. **P* < 0.05, ***P* < 0.01, ****P* < 0.001.

**Figure S3** (a) KEGG pathways from *Mu. leucogaster* in different sites based on LEfSe analysis. KEGG pathways with the highest linear discriminant analysis (LDA) scores.

**
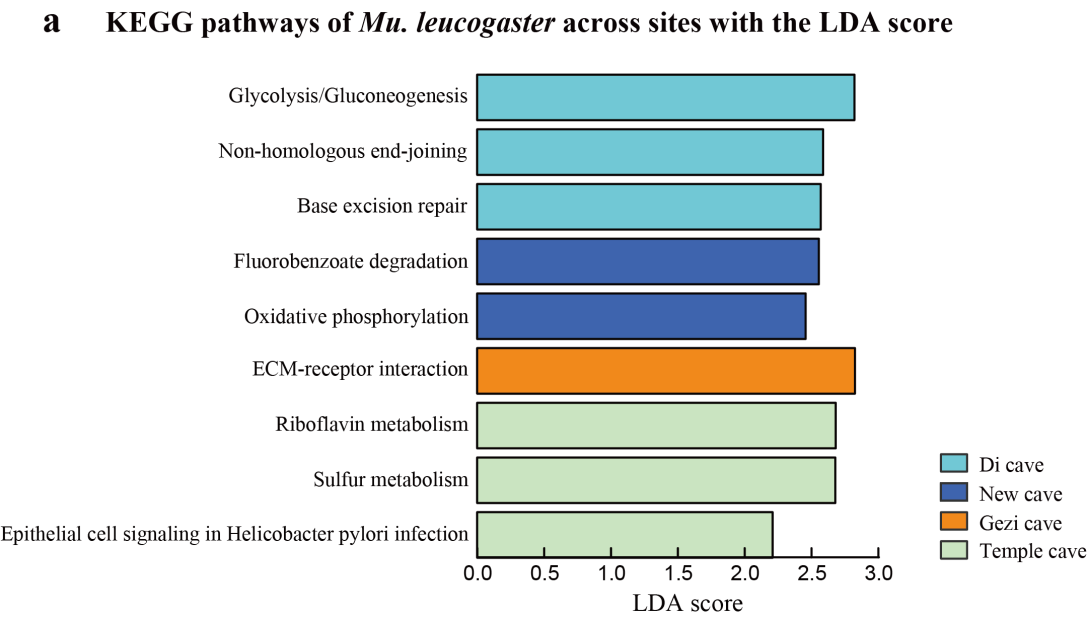
**

**Figure S4** (a) Gene abundance of pathways within metabolism of terpenoids and polyketides and biosynthesis of secondary metabolites from *Mu. leucogaster* across sites. The gene relative abundances of pathways > 0.1%. Asterisks indicate a significant difference among groups. **P* < 0.05, ***P* < 0.01, ****P* < 0.001.


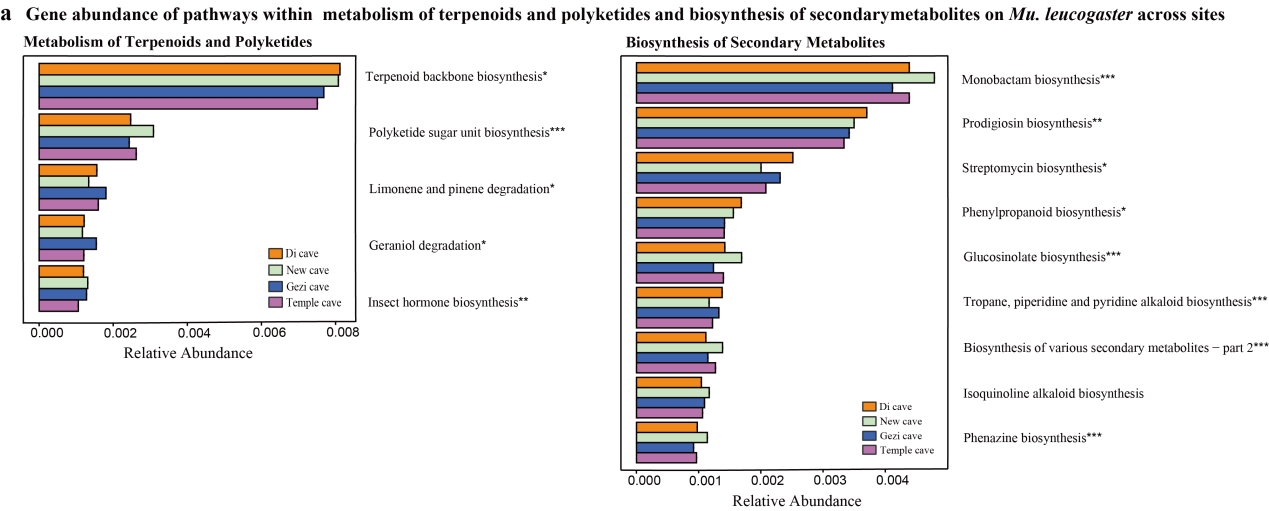

Supplement: Supplementary file 1 [file Data_Sheet_1.docx]
